# Induction of alopecia areata in C3H/HeJ mice using polyinosinic-polycytidylic acid (poly[I:C]) and interferon-gamma

**DOI:** 10.1038/s41598-018-30997-3

**Published:** 2018-08-21

**Authors:** Jung-Min Shin, Dae-Kyoung Choi, Kyung-Cheol Sohn, Jung-Woo Koh, Young Ho Lee, Young-Joon Seo, Chang Deok Kim, Jeung-Hoon Lee, Young Lee

**Affiliations:** 1 Department of Dermatology, School of Medicine, Chungnam National University, Daejeon, Korea; 2 Biomedical Research Institute, Chungnam National University Hospital, Daejeon, Korea; 3 Department of Anatomy, School of Medicine, Chungnam National University, Daejeon, Korea

## Abstract

Alopecia areata (AA) is a chronic, relapsing hair-loss disorder that is considered to be a T-cell-mediated autoimmune disease. Several animal models for AA have been created to investigate the pathophysiology and screen for effective therapeutic targets. As C3H/HeJ mice develop AA spontaneously in a low frequency, a novel animal model is needed to establish an AA-like condition faster and more conveniently. In this study, we present a novel non-invasive AA rodent model that avoids skin or lymph-node cell transfer. We simply injected C3H/HeJ mice subcutaneously with interferon-gamma (IFNγ) along with polyinosinic:polycytidylic acid (poly[I:C]), a synthetic dsRNA, to initiate innate immunity via inflammasome activation. Approximately 80% of the IFNγ and poly(I:C) co-injected mice showed patchy AA lesions after 8 weeks. None of the mice displayed hair loss in the IFNγ or poly(I:C) solely injection group. Immunohistochemical staining of the AA lesions revealed increased infiltration of CD4^+^ and CD8^+^ cells infiltration around the hair follicles. IFNγ and poly(I:C) increased the expression of NLRP3, IL-1β, CXCL9, CXCL10, and CXCL11 in mouse skin. Taken together, these findings indicate a shorter and more convenient means of AA animal model induction and demonstrate that inflammasome-activated innate immunity is important in AA pathogenesis.

## Introduction

Alopecia areata (AA) is a cell-mediated autoimmune disease that targets anagen-stage hair follicles. Although AA is not a life-threatening disease, it may lead to psychological consequences, including high levels of anxiety and depression^1,2^. AA animal models are urgently needed to elucidate the pathogenesis and screen for effective therapeutic targets. The most well-established animal models are inbred C3H/HeJ mice, which develop AA-like hair loss spontaneously or after experimental induction^3–5^, as well as humanized mouse model with transplantations of human scalp skin followed by either autologous or allogenic peripheral blood mononuclear cells, to severe-combined immunodeficient (SCID) mice^6^. However, existing animal models have limitations including low frequency of occurrence of AA and difficulty in controlling AA onset and obtaining human scalp tissues.

T cells and a collapse of immune privilege of hair follicle play a critical role in initiating AA and Janus kinase pathway, NKG2D and its ligands are also identified as important factors in the pathogenesis of AA^5,6^ IFNγ is one of the key factors that lead to the collapse of immune privilege by upregulation of major histocompatibility complex in hair follicles and increase the occurrence rate of AA^7,8^. Intravenous injection of IFNγ-induced AA in young C3H/HeJ mice has been previously reported^9^; however, subcutaneous IFNγ injection failed to induce hair loss, suggesting that the route of administration and other co-factors are critical for induction of AA^10^.

Recently, we published a paper demonstrating the important role of innate immunity and inflammasome in AA pathogenesis. Polyinosinic-polycytidylic acid (poly[I:C]), a synthetic dsRNA, treatment-activated nucleotide binding oligomerization domain–like receptor family pyrin domain–containing 3 (NLRP3) inflammasome, toll-like receptor (TLR) 3, and Nuclear factor-κB (NF-κB) signaling result in tumor necrosis factor-α (TNF-α) and interleukin-1β (IL-1β) secretion in outer root sheath (ORS) cells^11^. To determine the role of inflammasome in the pathogenesis of AA in a mouse model, we used the spontaneous C3H/HeJ mouse model and injected recombinant murine interferon-gamma (IFNγ) along with poly(I:C) subcutaneously to trigger IL-1β-mediated inflammatory responses via the NF-κB pathway and inflammasome activation.

## Results

### IFNγ and poly(I:C)-induced AA in C3H/HeJ mice

**Figure 1:**
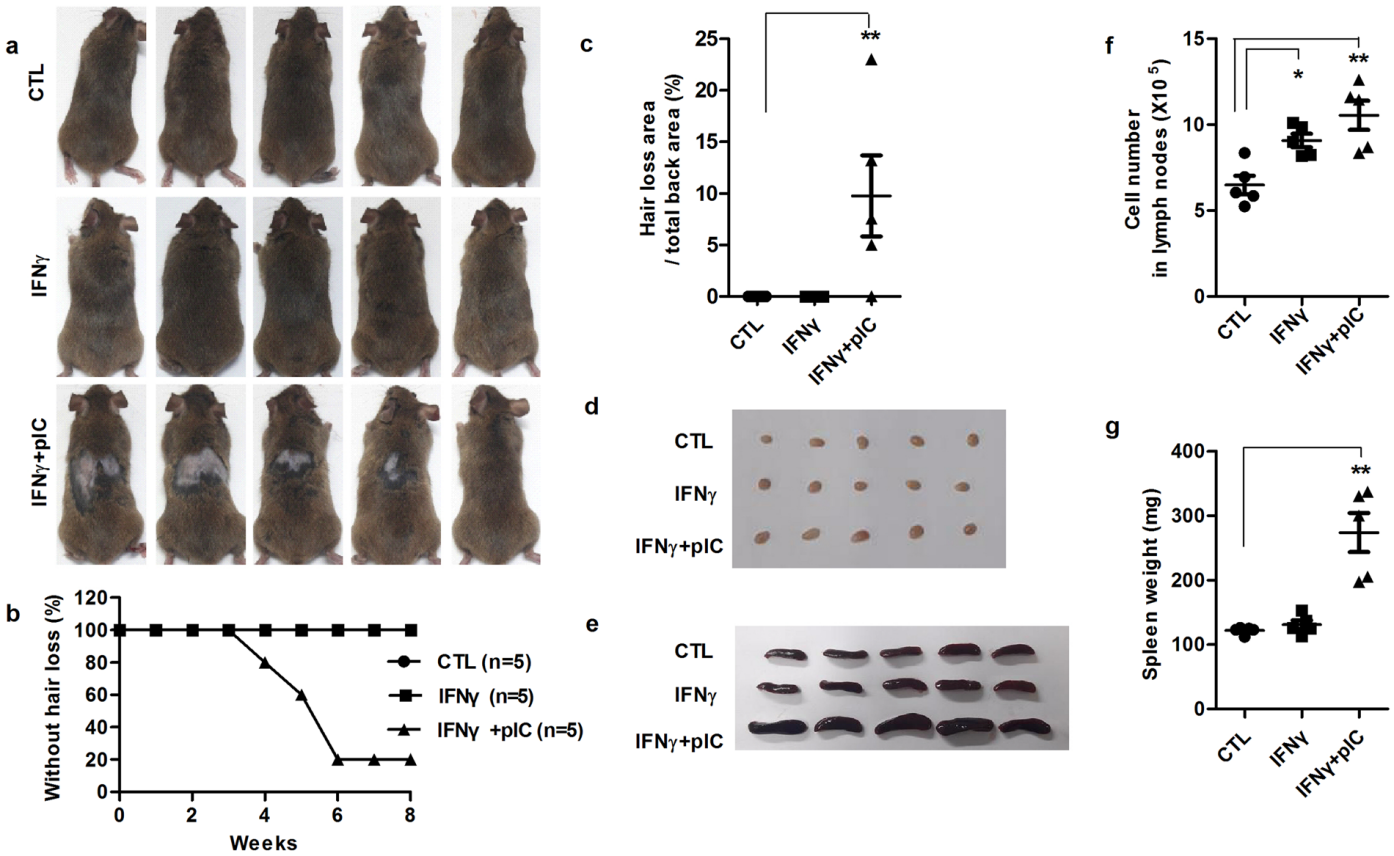
Development of alopecia areata induced by IFNγ and poly(I:C) in C3H/HeJ mice. Mice were subcutaneously injected with PBS (control), IFNγ only, IFNγ, and poly(I:C) together (IFNγ, 2 × 10^4^ units/mice, once/week; poly(I:C), 100 μg/mice, twice/week) for 8 weeks (n = 5 mice/group). (**a**) Hair loss in C3H/HeJ mice at 8 weeks after injection. (**b**) Time course of onset of AA after injection. (**c**) Hairless area in the back skin (**d**,**e**) Increased lymphadenopathy in IFNγ and poly(I:C) co-treated mice. (**f**,**g**) Increased number of isolated lymph node cells and spleen weight in IFNγ and poly(I:C) co-treated mice. Data were analyzed using one-way ANOVA (*p < 0.05, **p < 0.01).

We investigated whether co-treatment with IFNγ and poly(I:C) induces AA in C3H/HeJ mice. Fifteen-week-old mice were subcutaneously injected with IFNγ once and poly(I:C) twice weekly in dorsal area for 8 weeks. AA was observed in the dorsal skin at the IFNγ and poly(I:C) co-injection site. Approximately 80% of IFNγ and poly(I:C)-treated mice developed AA within 8 weeks; control and IFNγ-only injected mice showed no hair loss (Fig. 1a–c). We also found that affected mice showed lymphadenopathy with increased lymph node cell number and spleen weight (Fig. 1d–g). These results indicated that local injection of IFNγ and poly(I:C) lead to systemic inflammation, which is a well-known characteristic of an AA mouse model, in C3H/HeJ mice. For the preliminary study, we injected poly(I:C) (100 μg/mice) twice per a week for 8 weeks, however poly(I:C) solely injected group did not show AA lesion during 8 weeks of treatment (Supplemental Fig. S1).

**Figure 2:**
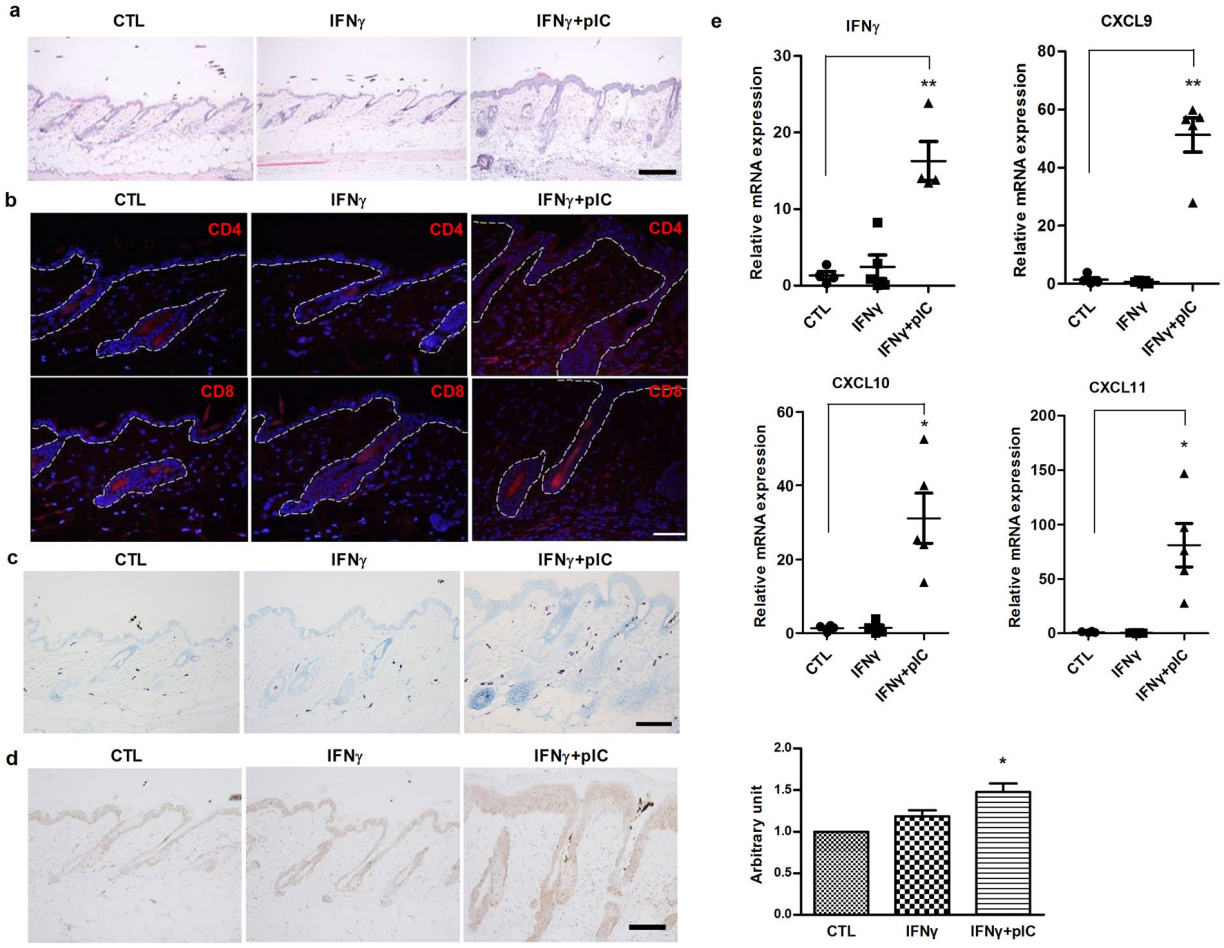
Histological changes of IFNγ and poly(I:C)-induced AA in C3H/HeJ mice. (**a**) Histological examination of the epidermis using hematoxylin and eosin (H&E)-stained skin sections at 8 weeks after injection. Bars = 100 μm. (**b**) Immunofluorescence staining of CD4 and CD8 in skin sections from C3H/HeJ mice. Arrows indicate hair follicles. (**c**) Detection of mast cells using toluidine blue staining in skin sections from C3H/HeJ mice. (**d**) Immunohistochemical staining of CXCL10 in skin sections from C3H/HeJ mice. Bars = 50 μm. (**e**) qPCR analysis of IFNγ, CXCL9-11 mRNA levels in whole skin lysates from C3H/HeJ mice. Data were analyzed using one-way ANOVA (*p < 0.05, **p < 0.01).

We examined histological changes in affected mice to further characterize the IFNγ and poly(I:C)-induced AA in C3H/HeJ mice. Similar to spontaneously developed AA, inflammatory infiltrates increased around the anagen hair follicles in the lesional skin of IFNγ and poly(I:C)-induced AA mice (Figs 2a and S2). Immunohistochemical staining showed that CD4^+^ and CD8^+^ cells significantly infiltrated the perifollicular and intrafollicular areas in IFNγ and poly(I:C)-induced AA mice (Fig. 2b). Mast cells and NKG2D+ cells were also increased around and along the hair follicles in the lesional skin of poly(I:C)-induced AA in C3H/HeJ mice (Figs 2c and S3).

Next, we examined the expression of IFN-inducible C-X-C motif chemokines (CXCL) 9–11, which have previously been implicated in directing Th1 inflammatory response of AA. Immunohistochemical staining showed that CXCL10 was significantly increased in ORS cells and inflammatory infiltrates around hair follicles (Fig. 2d). Moreover, qPCR showed increased expression of IFNγ and CXCL9-11 in the lesional skin of IFNγ and poly(I:C)-treated mice compared to control and IFNγ-only treated mice (Fig. 2e).

### NLRP3 inflammasome activation in IFNγ and poly(I:C)-induced AA mice

**Figure 3:**
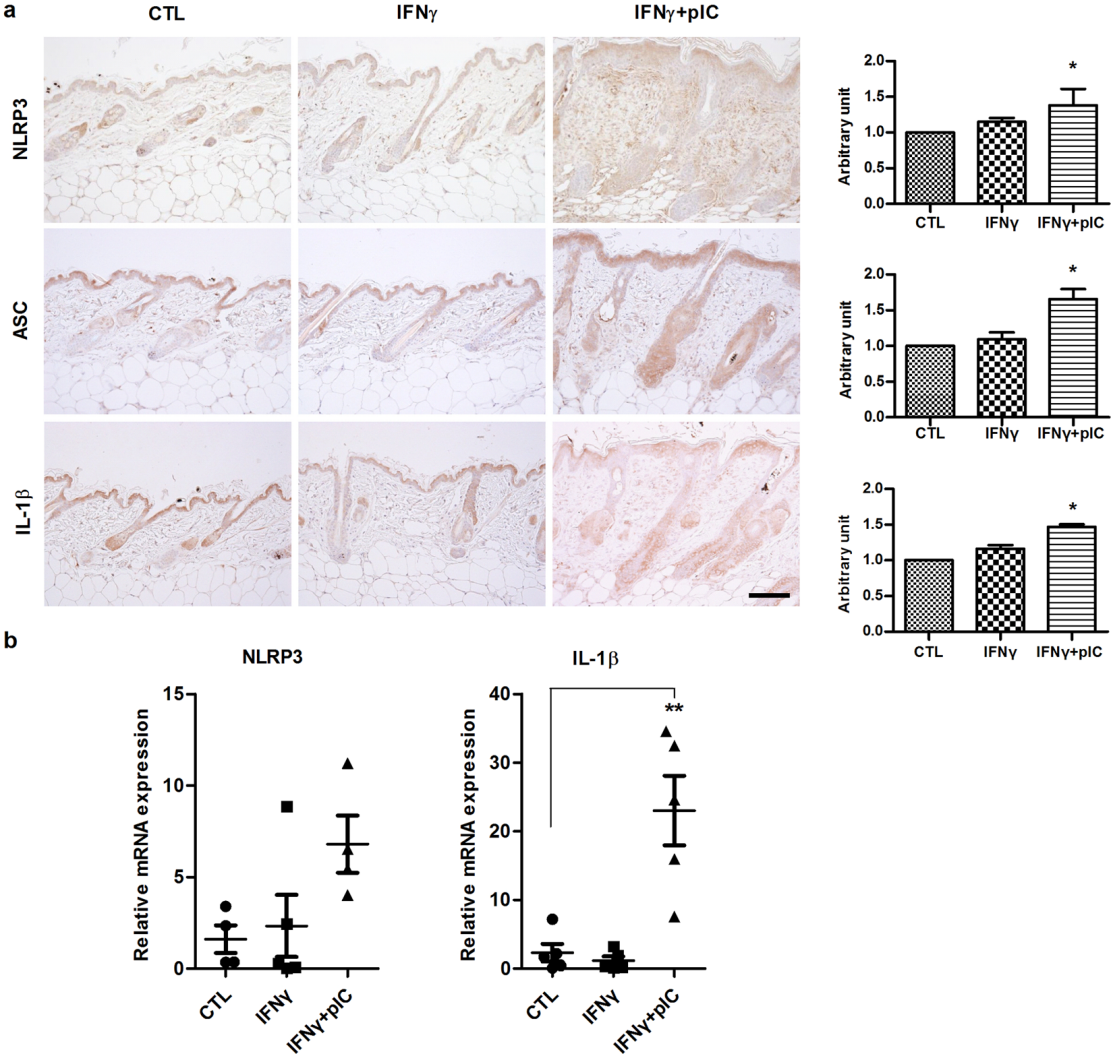
Activation of NLRP3 inflammasome in IFNγ and poly(I:C)-induced AA of C3H/HeJ mice. (**a**) Immunohistochemical staining of NLRP3, ASC, and IL-1β in skin sections from C3H/HeJ mice. Bars = 50 μm. (**b**) qPCR analysis of NLRP3 and IL-1β mRNA levels in whole skin lysates from C3H/HeJ mice. Data are presented as mean ± standard error of the mean (SEM). Data were analyzed by one-way ANOVA (*p < 0.05, **p < 0.01).

We previously reported that activation of NLRP3 inflammasome is involved in the pathogenesis of AA^11^. We determined whether IFNγ and pIC induce NLRP3 inflammasome activation in C3H/HeJ mice. Immunohistochemical staining showed that NLRP3, ASC, and IL-1β were weakly expressed in interfollicular epidermis in IFNγ-treated and control mice. However, the expressions were markedly increased in hair follicles and inflammatory infiltrates around hair follicles of IFNγ and poly(I:C)-treated mice (Fig. 3a). Surprisingly, the mouse which did not show hair loss after IFNγ and poly(I:C)-treatment also showed inflammatory cell infiltration around hair follicles and expressed NLRP3. However, there was weak expression of IL-1β and ASC compared to mice with hair loss (Supplemental Fig. S4). NLRP3 and IL-1β mRNAs were significantly upregulated in the lesional skin of IFNγ and poly(I:C) co-treated mice, compared with control and IFNγ-only treated mice (Fig. 3b). These results indicate that NLRP3 inflammasome activation may be associated with the pathogenesis of IFNγ and pIC-induced AA in C3H/HeJ mice.

### Effect of IFNγ and poly(I:C) on inflammatory reaction of ORS cells

**Figure 4:**
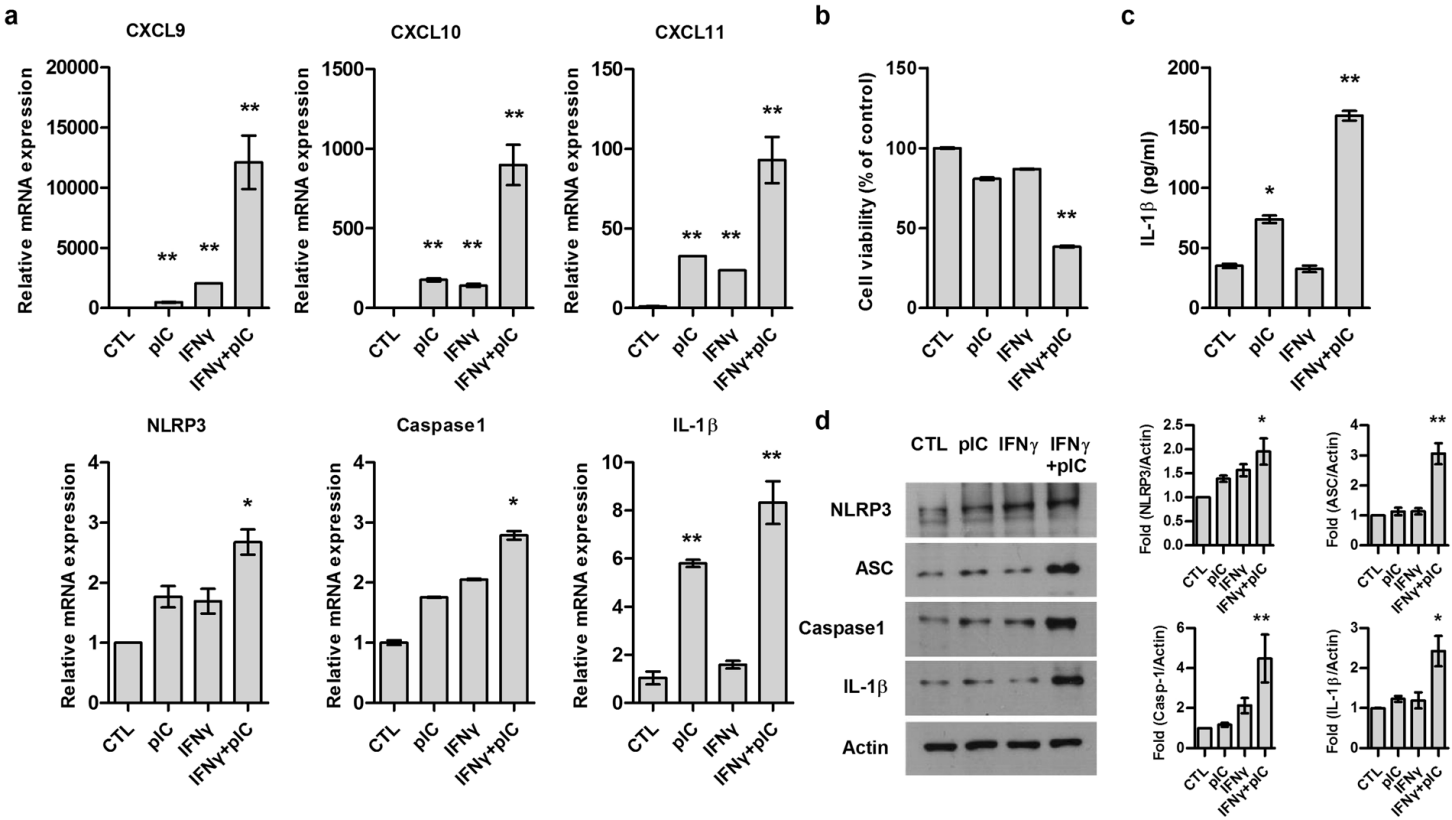
Effect of IFNγ and poly(I:C) co-treatment on inflammatory response in ORS cells. ORS cells were treated with IFNγ (5 ng/ml) or IFNγ and poly(I:C) (10 μg/ml) together. (**a**) Using qPCR, relative mRNA expressions of CXCL9-11, NLRP3, caspase-1, and IL-1β was determined 2 hrs after treatment. (**b**) Cell viability was examined by 3-(4,5-dimethylthiazol-2-yl)-2,5-diphenyltetrazolium bromide (MTT) assay 24 hrs after treatment. (**c**) Secreted IL-1β was measured by enzyme-linked immunosorbent assay (ELISA) kit 24 hrs after treatment. (**d**) Expression levels of NLRP3, pro-caspase-1, ASC and IL-1β in the cell lysates were assessed after 48 hours by Western blotting. Actin was used as a loading control. Data are presented as mean ± standard error of the mean (SEM). Data were analyzed by one-way ANOVA (*p < 0.05, **p < 0.01). Cropped blots were used in this figure and full-length blots are presented in Supplemental Fig. 8.

We further investigated the effect of IFNγ and poly(I:C) on inflammatory responses *in vitro* with human ORS cells. Co-treatment of IFNγ and poly(I:C) synergistically upregulated the mRNA levels of CXCL9-11 and inflammasome components including NLRP3, caspase-1, and IL-1β when IFNγ and poly(I:C) were applied to ORS cells (Fig. 4a). Also, decreased cell viability and increased secretion of IL-1β were observed in IFNγ and poly(I:C) co-treated ORS cells using MTT and ELISA assay (Fig. 4b and c). We confirmed that NLRP3 inflammasome components such as NLRP3, caspase-1, ASC, and IL-1β were significantly increased when using co-treatment of IFNγ and poly(I:C) (Figs 4d and S8). Also, when normal human epidermal keratinocytes were co-treated with IFNγ and poly(I:C), the protein levels of NLRP3, ASC, and IL-1β were significantly increased (Supplemental Fig. S9). These results indicate that IFNγ and poly(I:C) induce pyroptosis by activating NLRP3 inflammasome in ORS cells. Furthermore, we examined whether IFNγ and poly(I:C) affect JAK-STAT signaling pathway, co-treatment of IFNγ and poly(I:C) increased phosphorylation of STAT3 on ORS cells (Supplemental Fig. S5).

## Discussion

Animal models play an important role in the investigation of human disease pathophysiology, target identification, as well as *in vivo* evaluation of therapeutic agents and treatment. For many years, several AA animal models have been established and greatly helped to elucidate cellular and molecular immune pathways in AA. The two most prominent ones are C3H/HeJ inbred mouse models and humanized AA mouse model.

The C3H/HeJ mouse models spontaneously develop AA-like hair loss phenotype up to 20% in aged ones^3^. However, this model has disadvantages including variation in incidence of AA occurrence and difficulty in controlling time of hair loss by the investigator. Several refined methods have been developed to generate a large number of AA-affected mice in a short period of time^4,5,12^. However, these methods still require lesional skin or lymph node cells of AA-affected mice.

The first humanized AA mouse model consisted of a lesional human AA scalp skin transplantation of onto SCID mice^13,14^. Recently a humanized model grafted healthy human scalp skin onto SCID mice, followed by intradermal injection of peripheral blood mononuclear cells, which had been cultured with high dose of IL-2^15^. The humanized model clinically reflects human disease pathology, but requires a substantial amount of human donor tissue. Thus, a new animal model with high frequency of occurrence and convenience in controlling disease onset is needed to investigate pathophysiology and screen the possible therapeutic molecules for AA.

There is a classical upregulation of IFNγ, TNF-α, and IL-1β in AA lesions^16,17^. In addition, a crucial role of IFNγ in AA pathogenesis has been confirmed in C3H/HeJ mice and it should be noted that IFNγ deficient mice are also resistant to AA^8,9^. However, IFNγ-induced AA in C3H/HeJ mice has been difficult to replicate presenting the role of other co-stimulatory signals to initiate the disease^10^.

Several studies have recently demonstrated the association between inflammasome and autoinflammatory and autoimmune skin diseases such as vitiligo, psoriasis, and pyoderma gangrenosum^18,19^. We previously provided evidence that human ORS cells contain the necessary elements to form NLRP3 inflammasomes, and that dsRNA upregulates inflammasome element as well as induces IL-1β via NLRP3 inflammasome activation. Furthermore, inflammasome activation mainly depends on TLR activation with downstream NF-κB signaling resulting in increased production of TNF-α. These findings suggest that AA is an autoinflammatory disease linked to the hyperactivation of the innate immune system^11^.

**Figure 5:**
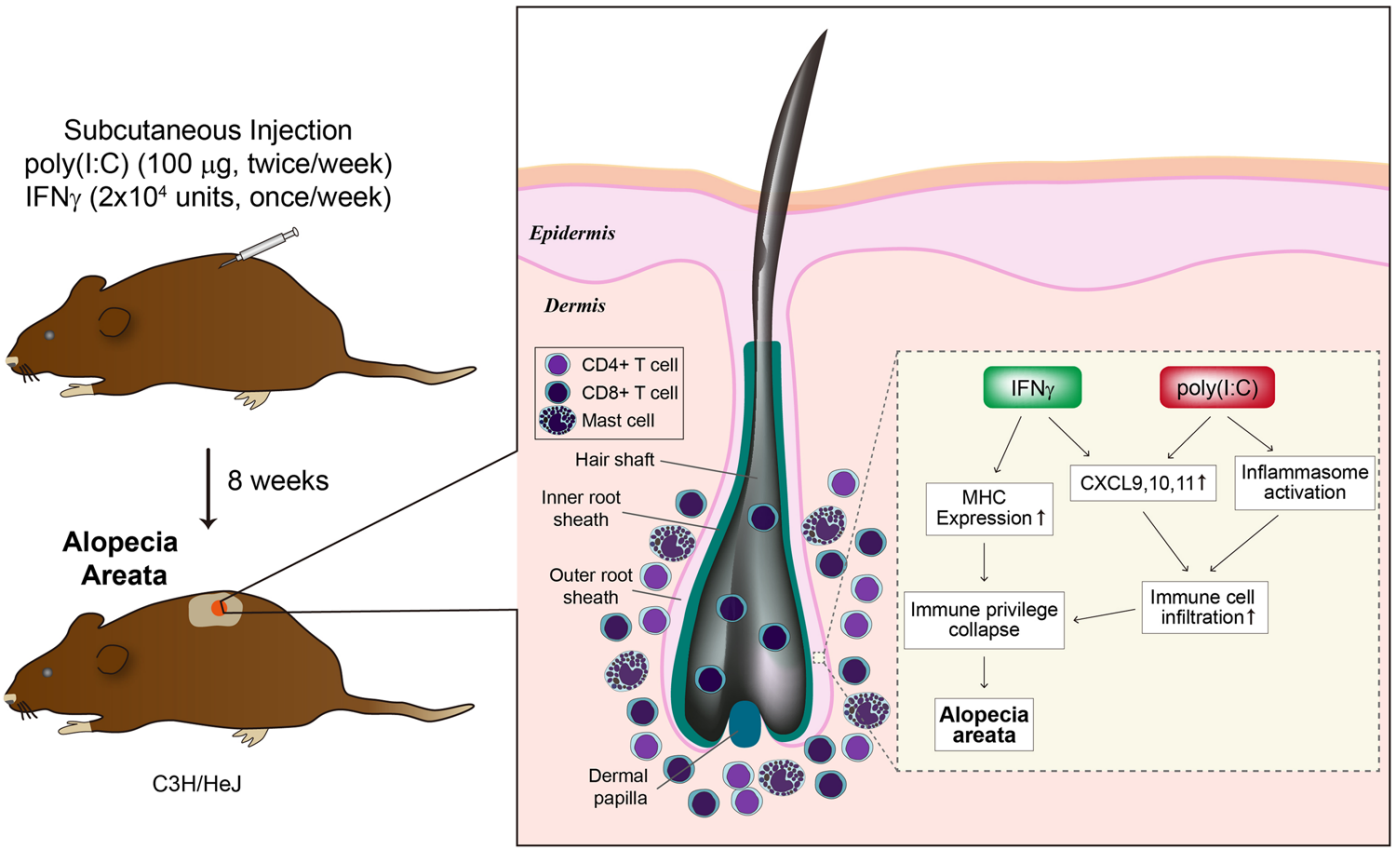
Summary of immunological changes in IFNγ and poly(I:C) induced AA in C3H/HeJ mice.

In this study, we used poly(I:C) to induce inflammasome in mouse skin to activate innate immunity and furthermore activate T-cell mediated immune responses. As we expected, subcutaneous co-injection of poly(I:C) and IFNγ induced hair loss in C3H/HeJ mice and neither poly(I:C) or IFNγ solely injected group showed AA lesion during 8 weeks of treatment. In poly(I:C) and IFNγ co-treated mice, there was marked infiltration of mast cells in dermis along with CD4^+^ and CD8^+^ T cells. This finding is consistent with human data showing that significantly greater physical mast cell/CD8^+^ T cell contact is observed, compared with healthy or non-lesional human skin. Furthermore, the mast cells are suggested to potentially present antigens and/or co-stimulatory signals to CD8^+^ T cells^20^. We also characterized the expression of IFN-inducible chemokines CXCL9, 10, and 11 and MHC class I and II (Supplemental Fig. S6). These expression signatures were well documented in previous reports of the lesional skin of AA animal models^21,22^. As expected, we observed marked upregulation of IFNγ and IFN-inducible chemokines in poly(I:C) and IFNγ–treated mice, compared with IFNγ treated mice. This finding demonstrates that poly(I:C) synergistically induces IFN-inducible chemokines with IFNγ and effectively induce inflammatory milieu in AA skin^23^. Moreover, there was an increased expression of NLRP3, ASC and IL-1β in poly(I:C) and IFNγ co-treated mouse skin, indicating that the activation of innate immunity via inflammasome in C3H/HeJ mouse could promote the development of AA in a genetically susceptible mouse strain with increase of MHC class I and II induced by IFNγ and poly(I:C) (Fig. 5). Interestingly, there was an increase of epidermal thickness in mice injected with poly(I:C) and IFNγ. We hypothesized it could be due to chronic inflammation induced by poly(I:C) and IFNγ and this finding is different from the human AA histopathology and the limits of this AA model. Further study is needed to elucidate the mechanism of how poly(I:C) and IFNγ increase epidermal thickness in C3H/HeJ mice. We repeated the same experiment in another mouse strain (C57Bl/6) to confirm that the AA phenomenon is limited to C3H/HeJ strain. We subcutaneously injected in dorsal area with IFNγ (2 × 10^4^ units/mice) once and poly(I:C) (100 μg/mice) twice per a week in 15-week-female C57Bl/6 mice. Neither IFNγ, poly(I:C) or poly(I:C) and IFNγ co-injected mice show AA lesion during 8 weeks of treatment (Supplemental Fig. S7).

The current hypothesis used to explain AA pathogenesis focuses on immune privilege collapse in hair follicles and autoreactive lymphocytes activation^24^. In our mouse model, hair loss was only detected in IFNγ and poly(I:C) subcutaneous co-injected young mice after 8 weeks. IFNγ induced expression of MHC on follicular epithelium and IFN-inducible chemokines^7,9^ and potentially, poly(I:C) must have boosted the inflammation by inducing inflammasome complex in mouse skin resulting in innate immune system activation and further IFN-inducible chemokines and T cell-mediated immune responses induction. In addition to the inflammatory lymphocyte infiltration, our *in vitro* data suggest that the role of inflamed hair follicle keratinocytes may also contribute to AA immune response by releasing chemokines such as CXCL9, CXCL10, and CXCL11 and IL-1β cytokine.

In conclusion, we developed an AA mouse model using IFNγ and poly(I:C) subcutaneous co-injection into normal haired C3H/HeJ mice. This novel mouse model provides shorter and more convenient methods of AA induction and may be used as a preclinical screening device for various future therapeutic candidate molecules. This new mouse model also provides evidence that IFNγ needs co-stimulatory signal to initiate disease and demonstrates that NLRP3 inflammasome-mediated activation of innate immunity is important in the pathogenesis of AA. Further studies are warranted to investigate inflammasome signaling dysregulation in patients with AA and the development of possible treatment targets for AA via modulation of inflammasome pathway inhibition.

## Materials and Methods

### Mice

15-week-old female C3H/HeJ mice were obtained from the Central Lab. Animal Inc. (Seoul, Korea). For 8 weeks, mice were subcutaneously injected in dorsal area with IFNγ (2 × 10^4^ units/mice) once and poly(I:C) (100 μg/mice) twice per a week. The onset of hair loss was examined daily and documented. Lymph nodes were passaged through sterile cell strainer with DMEM medium. Isolated cells were washed by centrifugation at 1000 rpm for 5 min and resuspended in fresh medium. The number of lymph node cells was measured by cell counter. All experiments were performed in accordance with institutional guidelines and approved by Chungnam National University institutional animal care and use committee (IRB CNU-00733).

### ORS cell culture

Human scalp tissues were obtained under the written informed consent of donors, in accordance with the ethical committee approval process of the Institutional Review Board of Chungnam National University Hospital (IRB No. 1011–135). Hair follicles were isolated from scalp specimens according to a previously reported method (Sohn K. C. *et al*., 2009). Hair follicles were incubated with 0.25% trypsin, 0.02% ethylenediaminetetraacetic acid (EDTA) in phosphate-buffered saline (PBS) for 10 min. Hair follicles were then vigorously pipetted to obtain single cell populations. The dissociated cells were rinsed in Dulbecco’s modified Eagle’s medium (HyClone, Logan, UT, USA) supplemented with 10% fetal bovine serum (Gibco, Grand Island, NY, USA), and centrifuged for 5 min at 200 g. ORS cells were then resuspended in keratinocyte-serum free medium (K-SFM) supplemented with epidermal growth factor (EGF) and bovine pituitary extract (Gibco) and seeded onto culture dish. Cultures were maintained at 37 °C in a humidified atmosphere of 5% CO_2_.

### Reagents

Poly(I:C) was obtained from InvivoGen (San Diego, CA). Recombinant murine IFNγ was obtained from Peprotech (315-05, Rocky Hill, NJ). For immunohistochemical analysis and western blotting, we used the following specific antibodies: CD4 and CD8 (Biolegend, San Diego, CA), CXCL10 (R&D systems, Minneapolis, MN), IL-1β (Abcam, Cambridge, MA), caspase-1 (Cell Signaling Technology, Danvers, MA), NLRP3 and ASC (Adipogen, San Diego, CA), NKG2D (Abcam, Cambridge, MA) and actin (Santa Cruz Biotechnologies, Santa Cruz, CA).

### Immunohistochemistry

Tissue samples were fixed with 10% formaldehyde, embedded in paraffin, and cut into 4-μm-thick sections. The sections were deparaffinized in xylene and then rehydrated using an alcohol series. The primary antibody was diluted 1:100 and was incubated at 4 °C for overnights. The sections were then incubated with secondary antibody at room temperature for 30 minutes. The sections were incubated with diaminobenzidine tetrachloride solution at room temperature for 1 minute and counterstained with Mayer’s hematoxylin. For immunofluorescence, the sections were incubated with Alexa Fluor dyes-conjugated secondary antibodies after incubation of primary antibody and counterstained with 4′,6-Diamidine-2′-phenylindole dihydrochloride (DAPI). And they were finally visualized under a fluorescence microscope (Olympus Corporation, Tokyo, Japan). The intensity of ASC, NLRP3, IL-1β and CXCL10 was analyzed using ImageJ analysis program (http://imagej.nih.gov/ij/docs/index.html).

### MTT assay

Cells were treated with 5 mg/ml MTT (3-(4,5-dimethylthiazol-2-yl)-2,5-diphenyltetrazolium bromide) solution and were incubated for a further 30 min. The medium was removed and the resulting formazan crystal was solubilized in dimethylsulfoxide (DMSO). The optical density at 540 nm was determined using an ELISA reader.

### ELISA

Culture medium was collected 24 hours after treatment of IFNγ and/or poly(I:C), and secreted IL-1β was determined using commercial ELISA kits purchased from R&D Systems (Minneapolis, MN).

### Quantitative real-time polymerase chain reaction (qPCR)

Total RNAs from mice lesional skin and human ORS cells were isolated using Easy-blue RNA extraction kit (Intron, Daejeon, Korea). Human ORS cells were treated with IFNγ (5 ng/ml) or IFNγ and poly(I:C) (10 μg/ml) together and relative mRNA expressions of CXCL9-11, NLRP3, caspase-1, and IL-1β was determined 2 hrs after treatment. Two μg of total RNAs were reverse transcribed with moloney-murine leukaemia virus (M-MLV) reverse transcriptase (RTase) (Elpis Biotech, Daejeon, Korea). Aliquots of RT mixture were amplified using SYBR Green real-time PCR master mix (Applied Biosystems, Waltham, MA). The following primers sequences were used: For mouse, IFNγ, 5′-TTGGCTTTGCAGCTCTTCCT-3′, and 5′-TGACTGTGCCGTGGCAGTA-3′; CXCL9, 5′-GTTCGAGGAACCCTAGTGATAAGG-3′ and 5′-CCTCGGCTGGTGCTGATG-3′; CXCL10, 5′-GATGACGGGCCAGTGAGAA-3′ and 5′-GCTCGCAGGGATGATTTCAA-3′; CXCL11, 5′-GCCCTGGCTGCGATCA-3′ and 5′-TGTTTGAACATAAGGAAGCCTTGA-3′; NLRP3, 5′-ACGTGGTTTCCTCCTTTTGTA-3′ and 5′-TGAAAAAAACCCAGGGAA-3′; IL-1β, 5′-AGTTGACGGACCCCAAAAGAT-3′ and 5′-GGACAGCCCAGGTCAAAGG-3′. For human, CXCL9, 5′-GCAAGGAACCCCAGTAGTGAGA-3′ and 5′-CCCTTGGTTGGTGCTGATG-3′; CXCL10, 5′-TTCCTGCAAGCCAATTTTGTC-3′ and 5′-TCTTCTCACCCTTCTTTTTCATTGT-3′; CXCL11, 5′-CAAGGCTTCCCCATGTTCA-3′ and 5′-GCTTTTACCCCAGGGCCTAT-3′; NLRP3, 5′-TCTGTGTGTGGGACTGAAGCA-3′ and 5′-CAGCTGACCAACCAGAGCTTCT-3′; Caspase-1, 5′-AAAAAATCTCACTGCTTCGGACAT-3′ and 5′-TCTGGGCGGTGTGCAAA-3′; IL-1β, 5′-TTAAAGCCCGCCTGACAG A-3′ and 5′-GCGAATGACAGAGGGTTTCTTAG-3′.

### Western blot analysis

Human ORS cells were treated with IFNγ and/or IFNγ plus poly(I:C). Two days after treatment, protein levels of NLRP3, pro-caspase-1, ASC and proIL-1β were assessed. Cells were lysed in Proprep solution (Intron, Deajeon, Korea). Total protein was measured using a BCA protein assay kit (Pierce Biotechnology, Rockford, IL). Samples were run on SDS-polyacrylamide gels, transferred onto nitrocellulose membranes, and incubated with appropriate primary antibodies. Blots were then incubated with peroxidase-conjugated secondary antibodies and visualized by enhanced chemiluminescence (Translab, Deajeon, Korea).

### Statistical analysis

All experiments were repeated at least three times with separate batches of cells. One-way analysis of variance was used to compare variances (ANOVA) within and among groups. Data were evaluated statistically using post hoc two-tailed Student’s t-tests. Statistical significance was set at p < 0.05.

## Electronic supplementary material

Supplementary Information
