# Supplementary material for: Induction of alopecia areata in C3H/HeJ mice using polyinosinic-polycytidylic acid (poly[I:C]) and interferon-gamma
Source: Sci Rep. 2018 Aug 21;8:12518. doi: 10.1038/s41598-018-30997-3 (PMC6104095; doi:10.1038/s41598-018-30997-3)
Supplement: Supplementary file 1 — Supplementary Information [file 41598_2018_30997_MOESM1_ESM.pdf]

Supplementary Information

Induction of alopecia areata in C3H/HeJ mice using polyinosinic-polycytidylic acid (poly[I:C]) and interferon-gamma

Jung-Min Shin[1], Dae-Kyoung Choi[2], Kyung-Cheol Sohn[1], Jung-Woo Koh[1], Young Ho Lee[3], Young-Joon Seo[1], Chang Deok Kim[1], Jeung-Hoon Lee[1], Young Lee[1*]

[1]Department of Dermatology, School of Medicine, Chungnam National University, Daejeon, Korea
[2]Biomedical Research Institute, Chungnam National University Hospital, Daejeon, Korea
[3]Department of Anatomy, School of Medicine, Chungnam National University, Daejeon, Korea

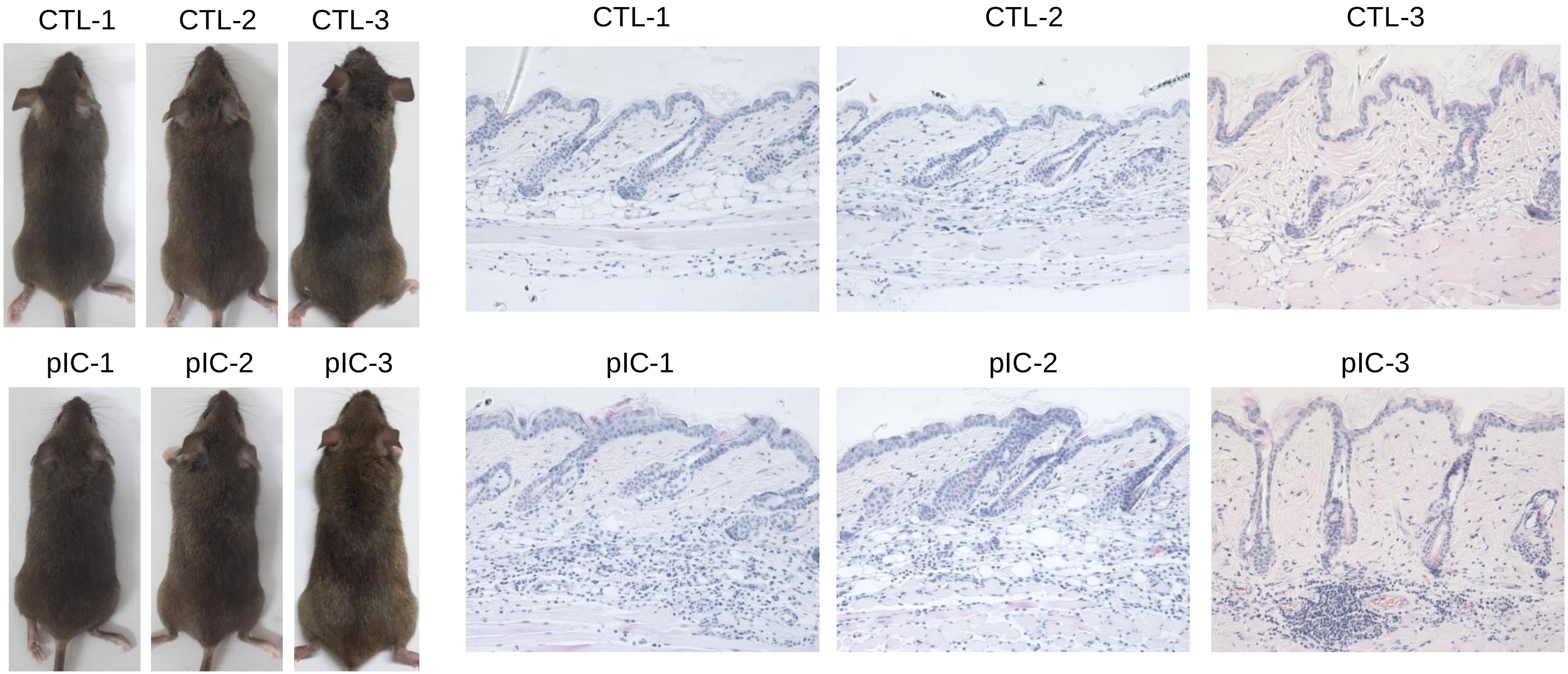

Supplemental Figure 1: 15-week-old female C3H/HeJ mice were subcutaneously injected in dorsal area with poly(I:C) (100 μg/mice) twice per a week for 8 weeks. Poly(I:C) solely injected group did not showed AA lesion during 8 weeks of treatment.

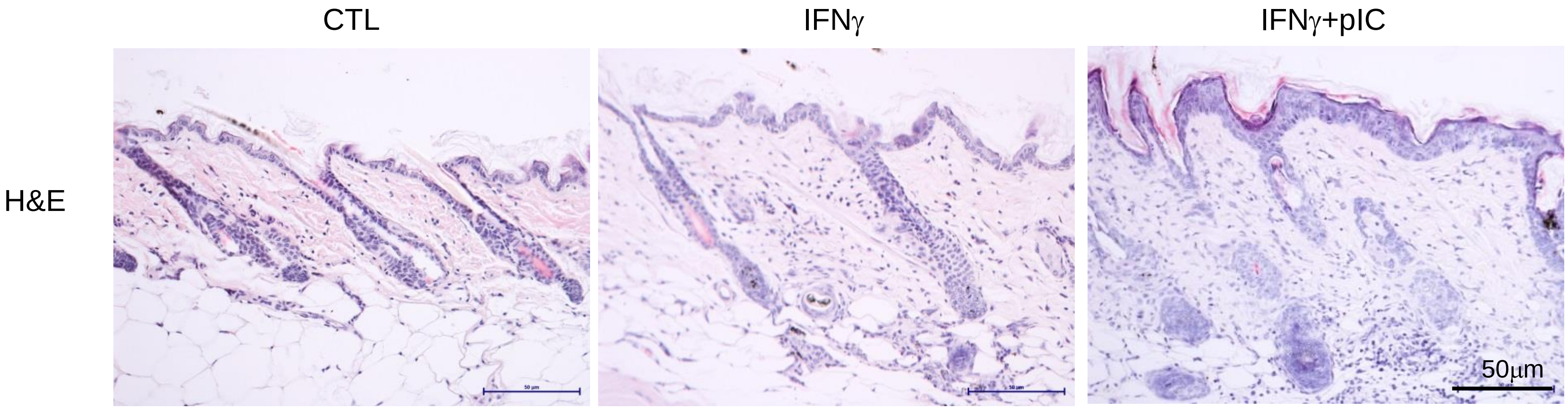

Supplemental Figure 2: Histological examination of PBS, IFNγ only, IFNγ and poly(I:C) treated C3H/HeJ mice at 8 weeks after injection at higher magnification (hematoxylin and eosin stain, bar = 50μm).

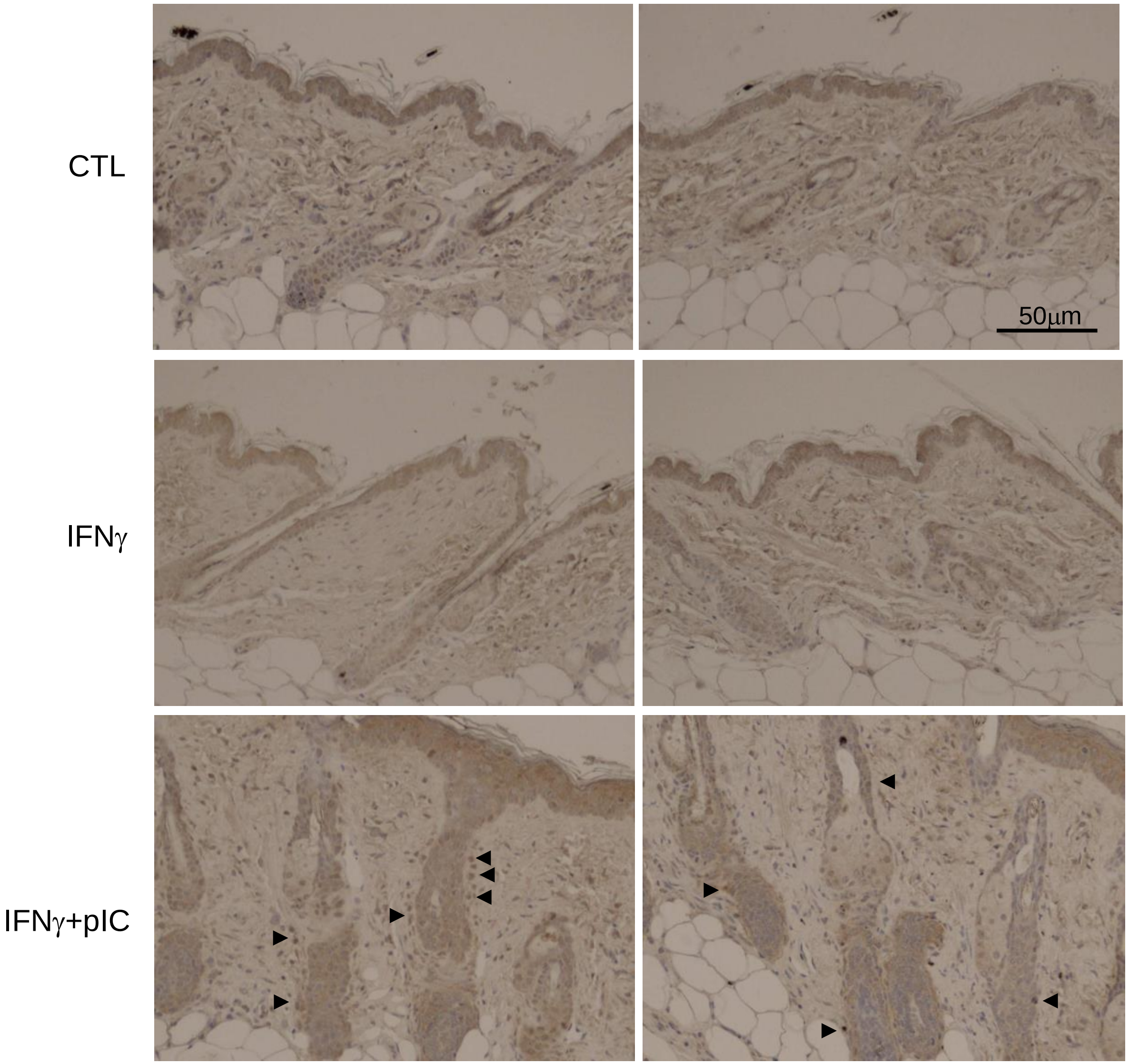

Supplemental Figure 3: Immunohistochemical staining of NKG2D in skin sections from PBS, IFNγ or IFNγ and poly(I:C) co-treated C3H/HeJ mice. NKG2D+ cells are detected around and along the hair follicles in IFNγ and poly(I:C) co-treated C3H/HeJ mice.

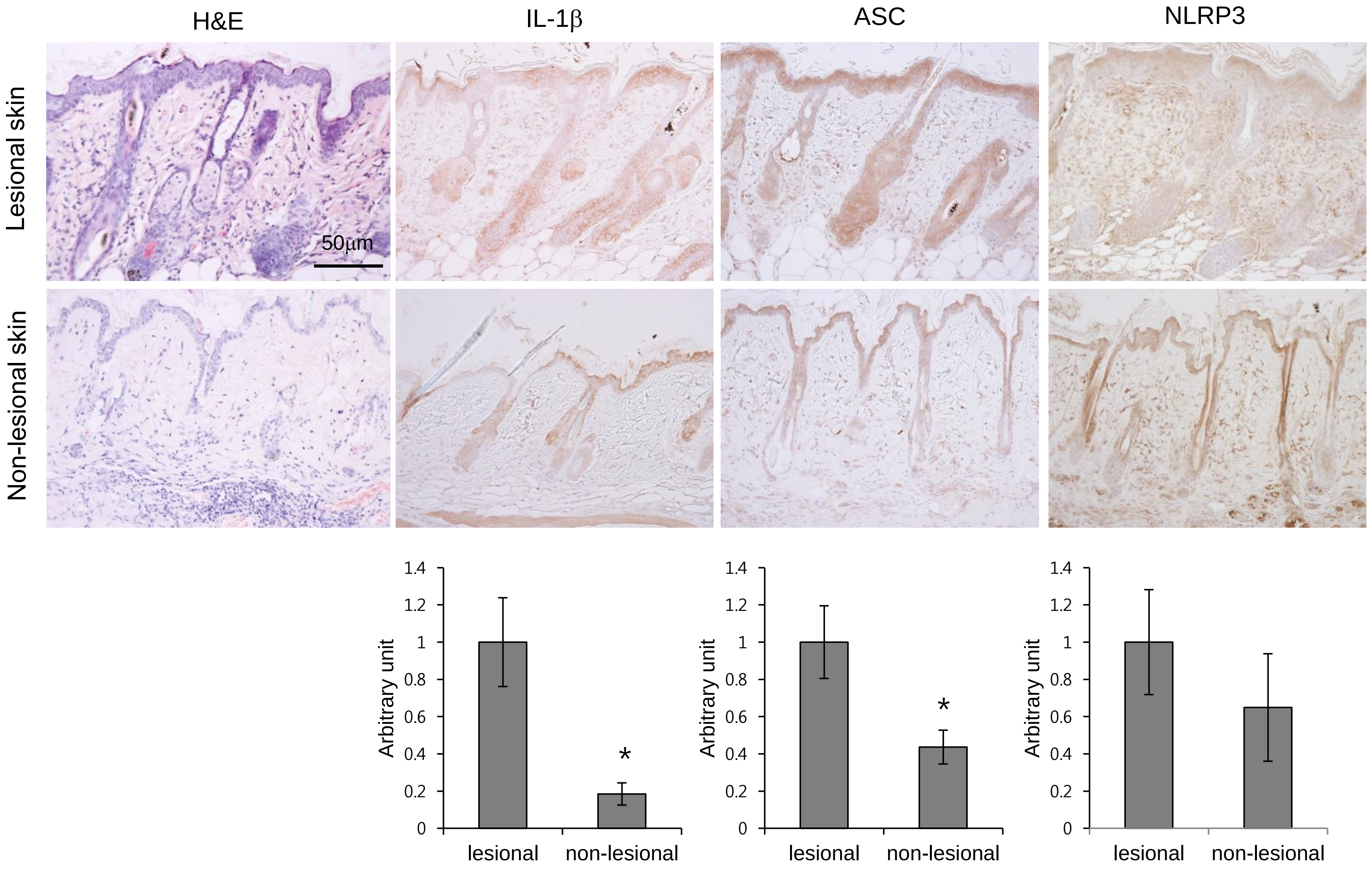

Supplemental Figure 4: Immunohistochemical staining of NLRP3, ASC, and IL-1β in skin sections from IFNγ and poly(I:C) co-treated C3H/HeJ mice without alopecia areata lesions.

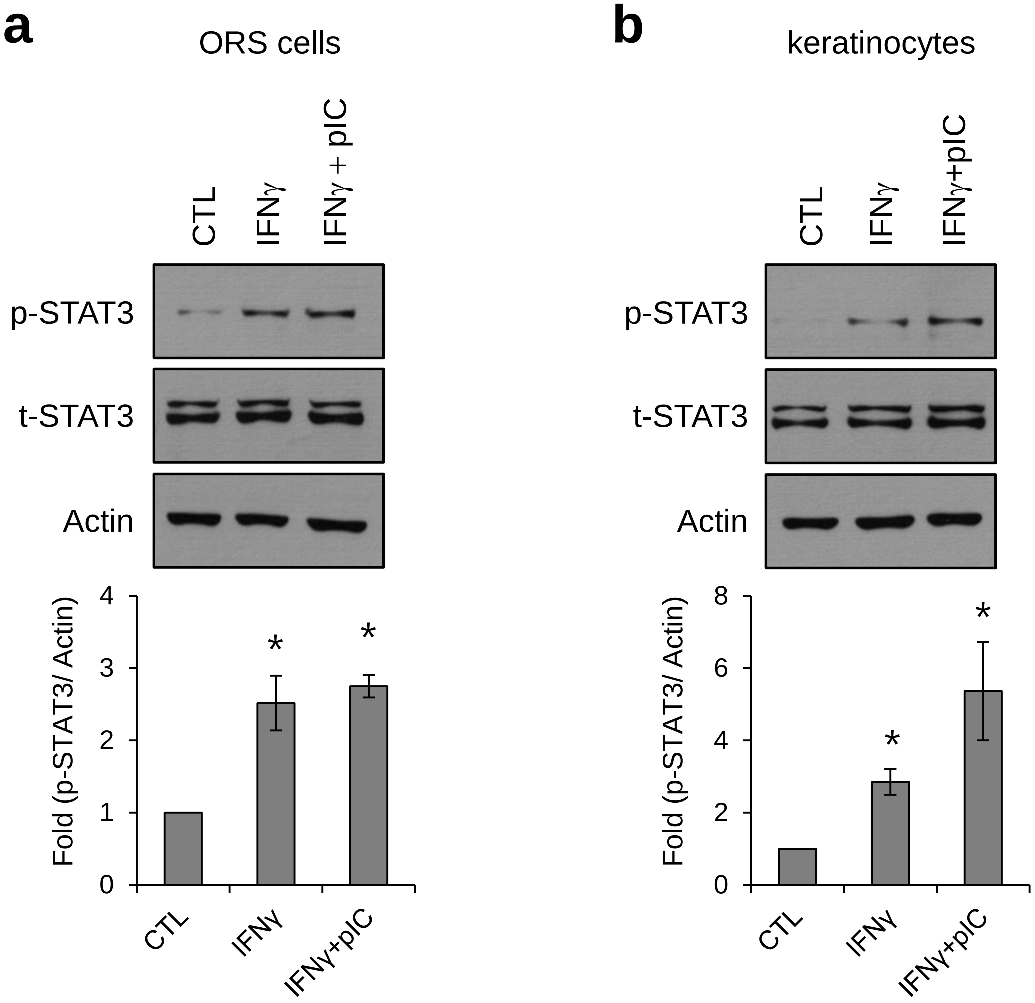

Supplemental Figure 5: ORS cells and keratinocytes were treated with interferon γ (IFNγ, 5 ng/ml) or IFNγ plus poly(I:C) (pIC, 10 μg/ml). Thirty minutes after treatment, protein levels of phosphorylated STAT3 (p-STAT3) and total STAT3 (t-STAT3) were assessed by Western blotting. Actin was used as a loading control. The p-STAT3 level was markedly increased in both the IFNγ-treated and IFNγ plus poly(I:C)-treated groups.

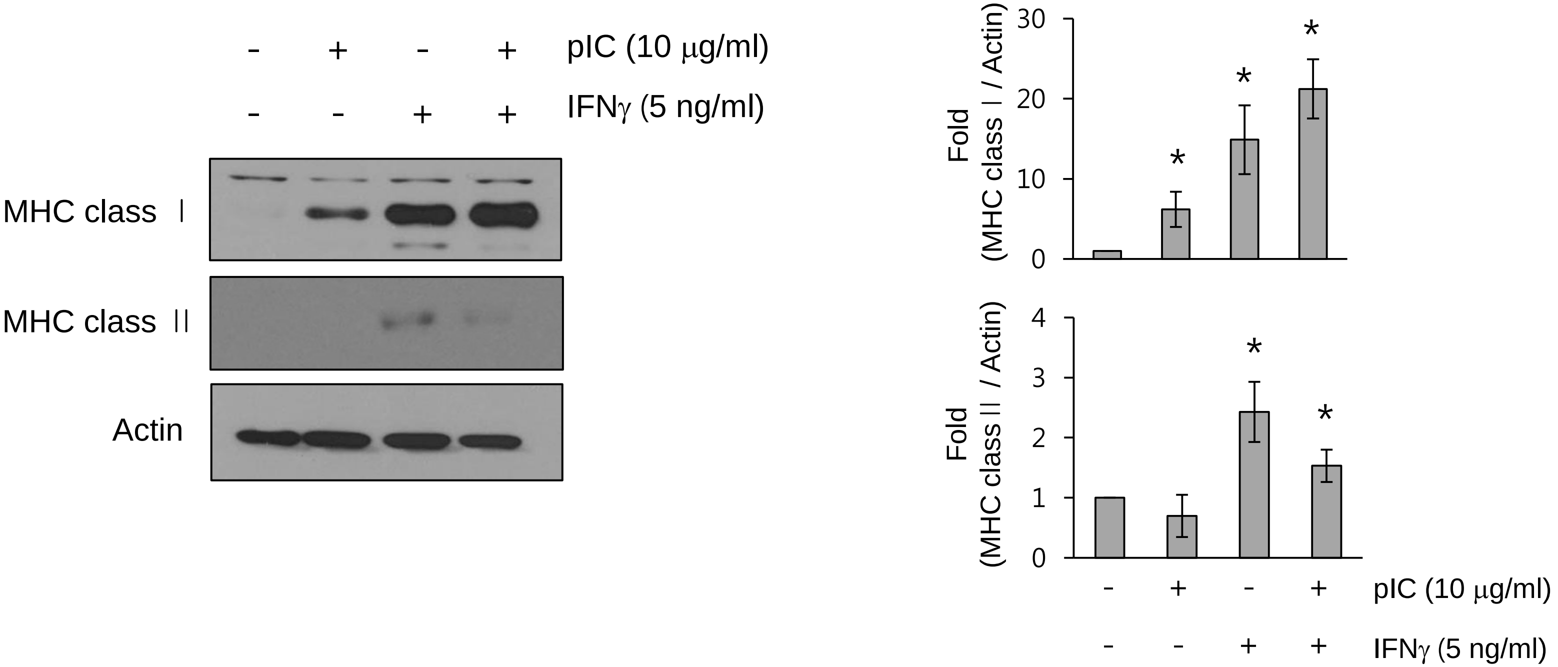

Supplemental Figure 6: ORS cells were treated with interferon γ (IFNγ, 5 ng/ml) or IFNγ plus poly(I:C) (pIC, 10 μg/ml). Two days after treatment, protein levels of MHC class I and MHC class II were assessed by Western blotting. Actin was used as a loading control. The MHC class I and MHC class II levels were markedly increased in both the IFNγ-treated and IFNγ plus poly(I:C)-treated groups.

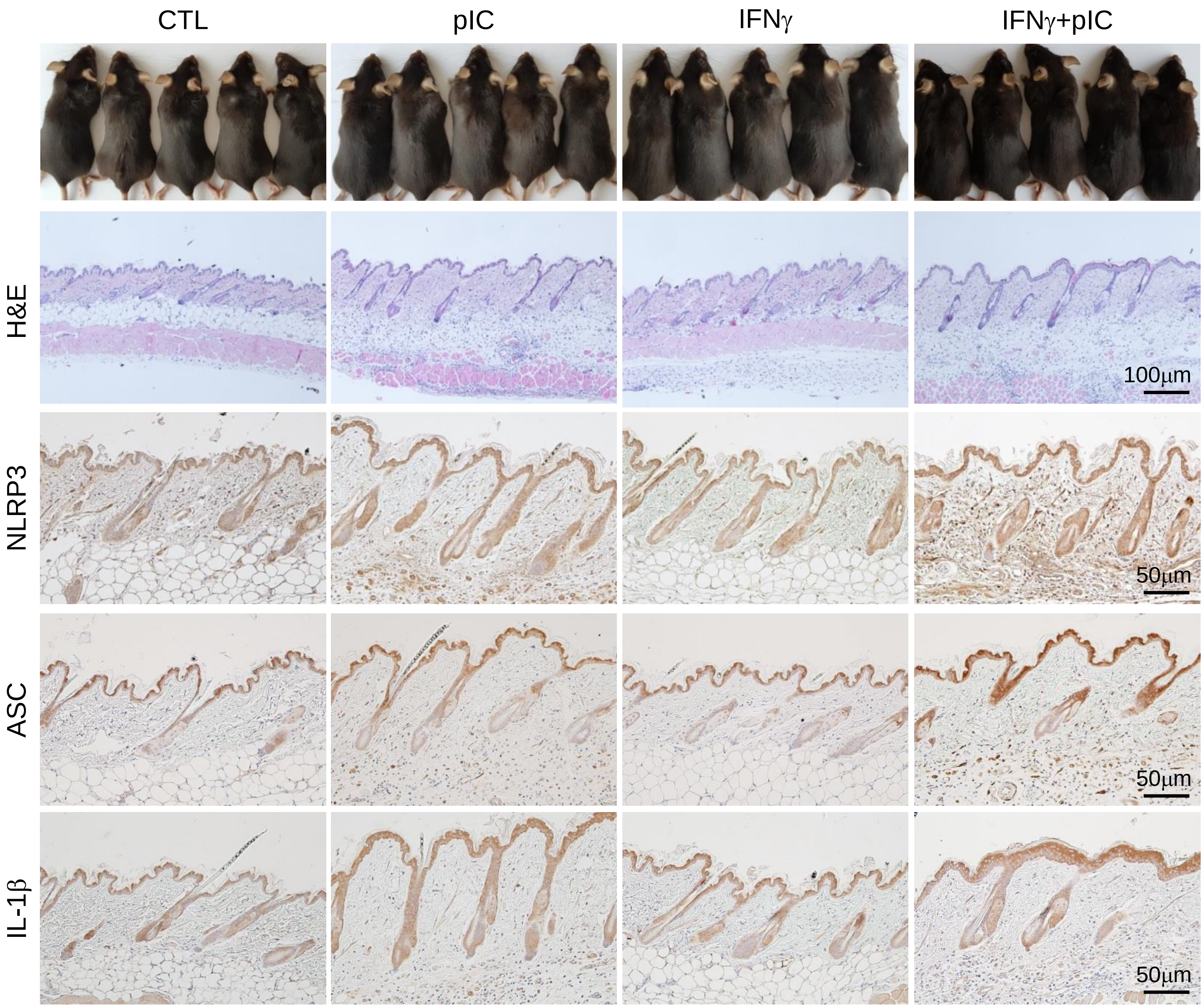

Supplemental Figure 7: 15-week-old female C57Bl/6 mice were subcutaneously injected in dorsal area with poly(I:C) (100 µg/mice, twice/week) and/or IFNg (2x10^4 units/mice, once/week) for 8 weeks. No hair loss was found in all groups, but the expression of NLRP3, ASC and IL-1β were increased by IFNγ plus poly(I:C) treatment.

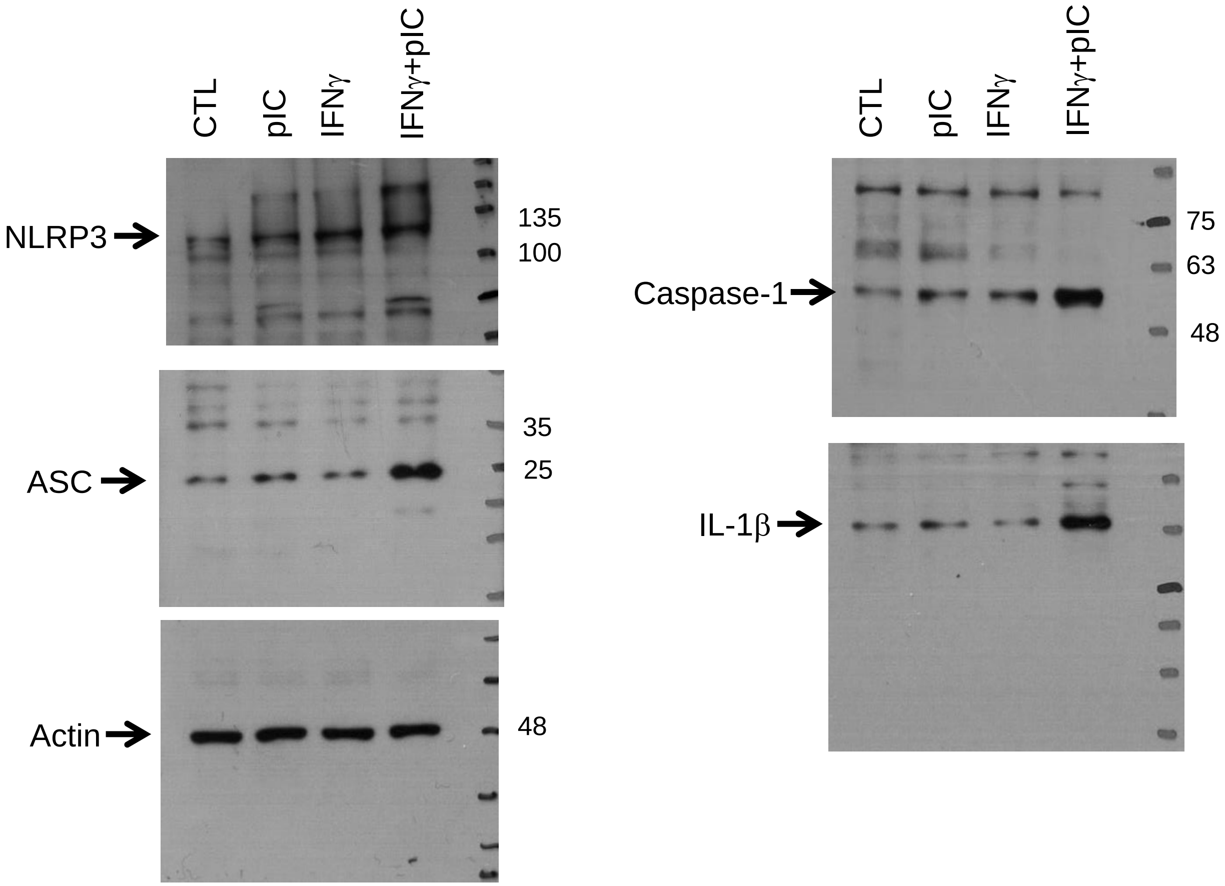

Supplemental Figure 8. Uncropped westernblots from supplemental figure 4d.

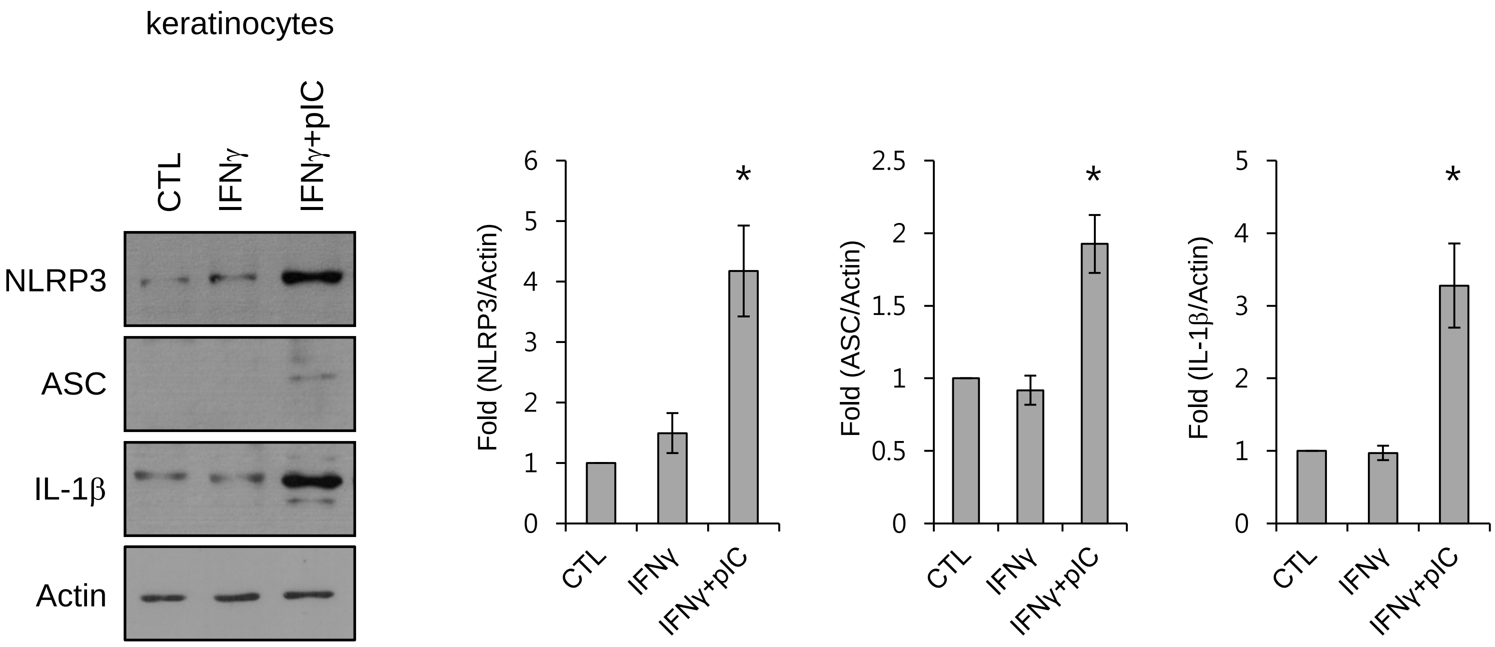

Supplemental Figure 9: Skin keratinocytes were treated with interferon γ (IFNγ, 5 ng/ml) or IFNγ plus poly(I:C) (pIC, 10 μg/ml). Twenty four hours after treatment, protein levels of NLRP3, ASC and IL-1β in the cell lysates were assessed by Western blotting. Actin was used as a loading control. The protein levels of NLRP3, ASC, and IL-1β were significantly increased when co-treated with IFNγ and poly(I:C) in keratinocytes.

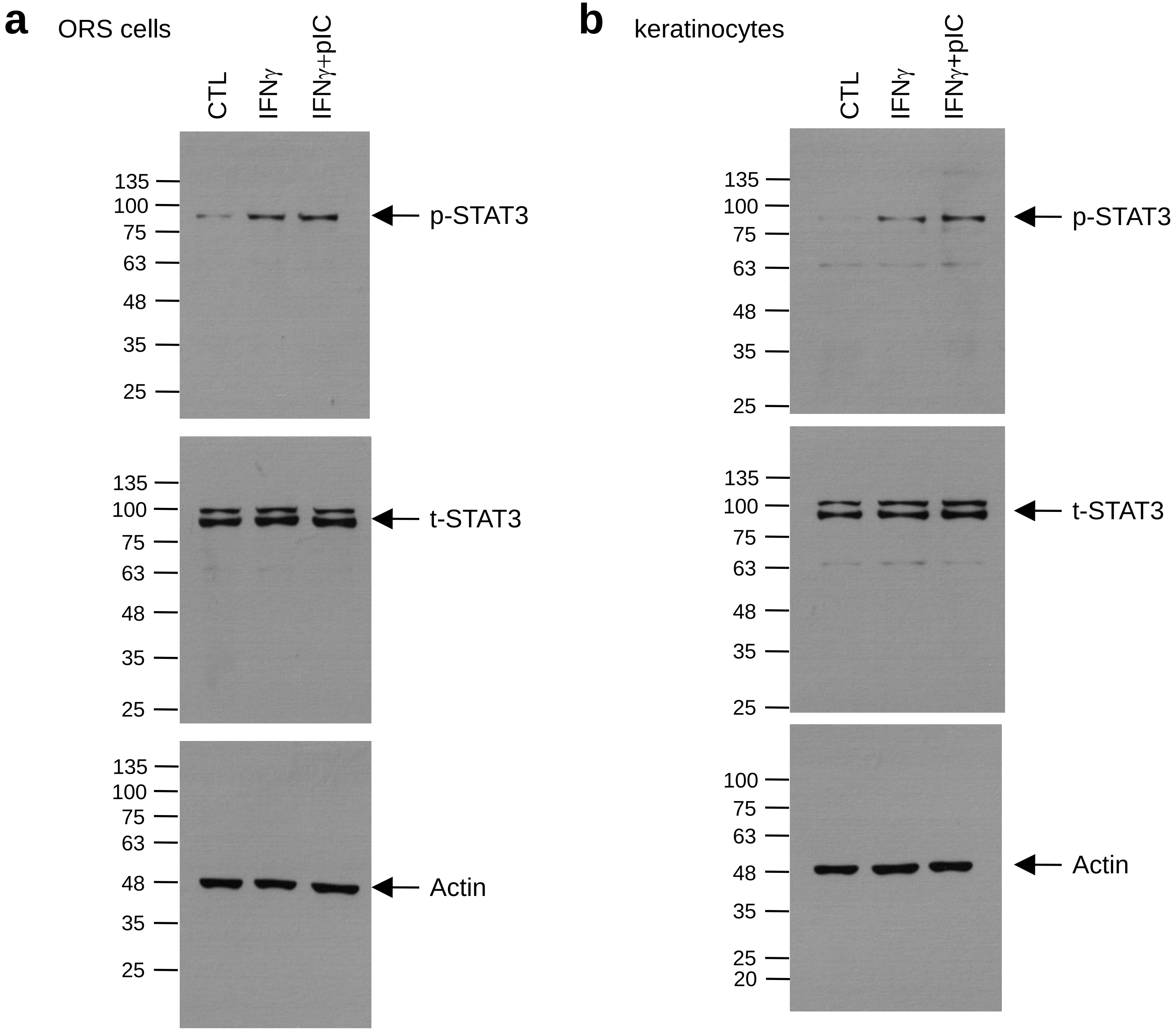

. Uncropped western blots from Supplemental Figure 5.

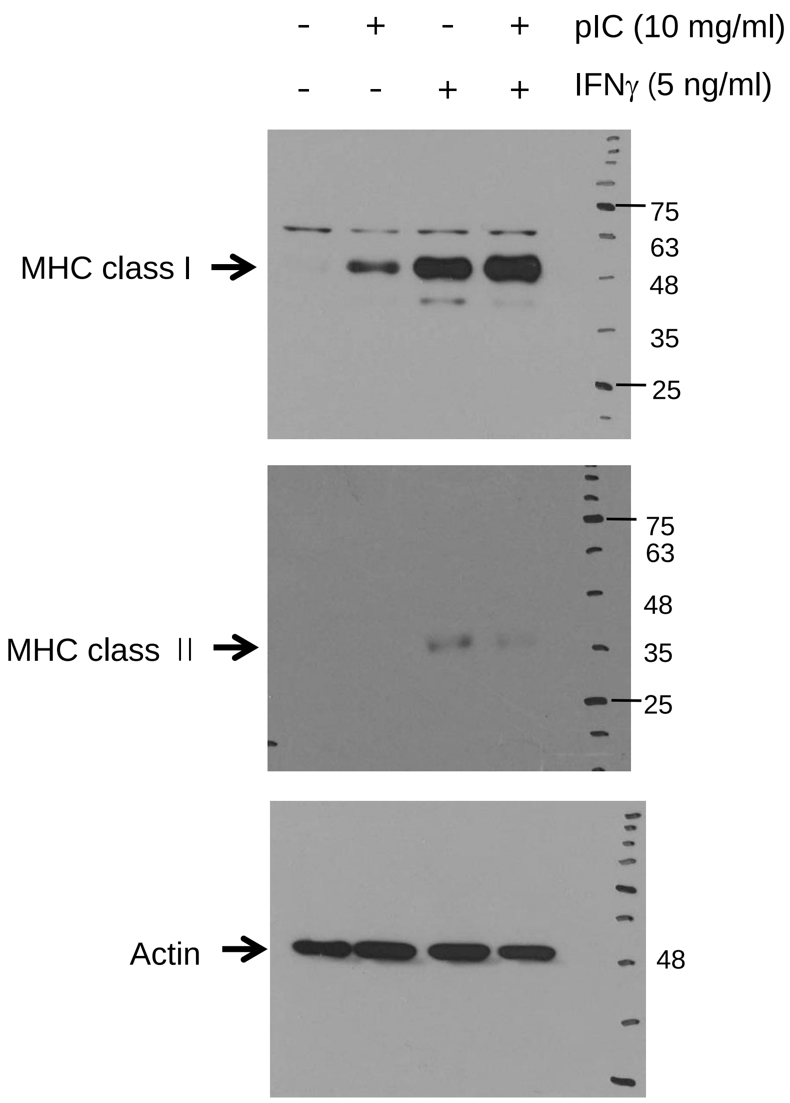

. Uncropped western blots from Supplemental Figure 6.

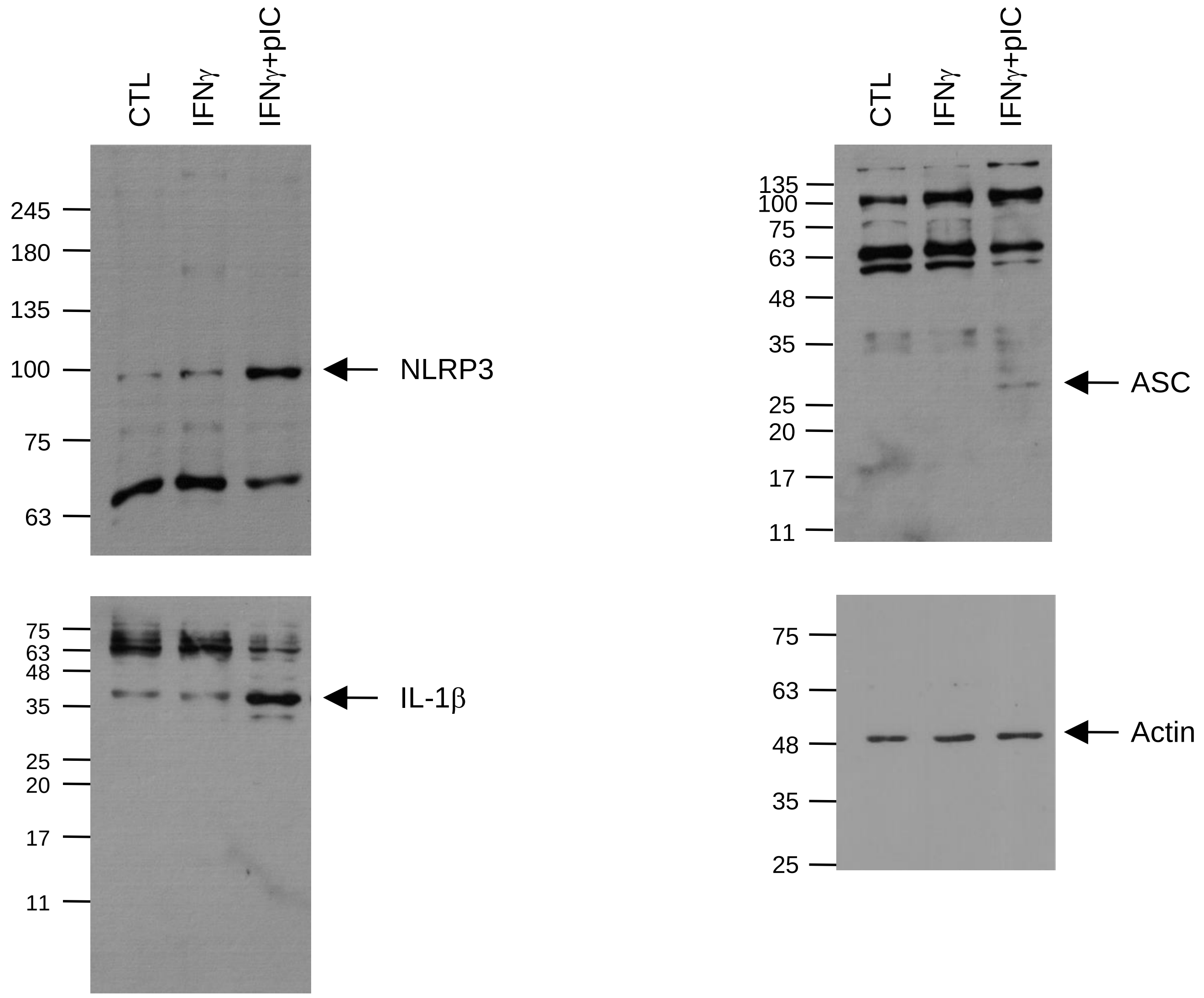

. Uncropped western blots from Supplemental Figure 8.
